# Solitary Skull Metastasis as the First Presentation of a Metachronous Primary Lung Cancer in a Survivor from Pancreatic Cancer

**DOI:** 10.1155/2017/5674749

**Published:** 2017-07-24

**Authors:** Ali Altalhy, Yazid Maghrabi, Zuhoor Almansouri, Saleh S. Baeesa

**Affiliations:** ^1^Division of Neurosurgery, Department of Surgery, Faculty of Medicine, King Abdulaziz University, Jeddah, Saudi Arabia; ^2^Department of Pathology and Laboratory Medicine, King Faisal Specialist Hospital and Research Center, Jeddah, Saudi Arabia

## Abstract

Skull metastasis from lung cancer is relatively common, yet the first presentation for this malignant disease is a rare occurrence. We herein report a case of a 54-year-old female, who had a good outcome following Whipple procedure for periampullary adenocarcinoma five years before her current presentation. During a routine follow-up, she was found to have a slowly progressive painless right parietal swelling. The systemic screening workup revealed no abdominal disease, but a solitary pulmonary nodule was identified. The presence of these two lesions raised the diagnosis of metastases from a previously treated pancreatic adenocarcinoma. The patient underwent complete excision of the skull lesion and subsequent lung biopsy, both of which proved on histopathological examination to be consistent with a primary lung cancer. This case emphasizes the importance of imaging and histopathological correlation in the diagnosis of solitary skull metastases and their effect on the subsequent management.

## 1. Introduction

Certain types of cancers such as carcinoma of the breast, lung, and kidney are associated with the highest rate of metastatic bone lesions, which approximately account for more than 75% of all patients affected by these types of cancers [1, 2]. It is known that many patients diagnosed with lung carcinoma are in an advanced stage of their disease at the time of diagnosis, and the presence of metastatic lesions is sign of poor prognosis of such cancer [1].

Bony metastases in lung carcinoma are the most common type of metastases associated with such malignant disease, accounting for 36% of affected patients [2]. Moreover, bone metastatic lesions are by far the most common reason for patients to seek medical advice since they result in severe pain [2]. Pain usually results from pathological fractures, which subsequently result from metastatic bony deposits [2].

A metachronous tumor is defined as the development of two primary malignant lesions usually with lag time, often years, between the first and second lesion [3]. Although rare, there have been many reports of metachronous pancreatic and lung adenocarcinoma [4–6]. Metachronous malignant lesions pose diagnostic dilemma since they are hard to differentiate from other metastatic lesions [4–6].

Skull metastases from breast, lung, and prostate carcinoma are relatively common accounting for 15–25% of all cancer patients [7, 8]. Primary malignancy in other sites such as renal, uterine, or hepatocellular sites has rarely been reported in the literature [9–12]. Skull metastasis is usually discovered as an incidental finding since it is asymptomatic in most cases [13]. Sometimes, metastatic lesions can produce mild pain or, with dural sinuses involvement, an increase in intracranial pressure (ICP), leading patients to seek medical advice [13].

Nevertheless, only one reported case in literature has presented a case of solitary skull mass as the first presentation of lung adenocarcinoma, in which a patient, with no history of a previous malignant disease, presented with a skull swelling that proved later to be the first presentation of lung malignancy [2]. Moreover, it differs from our current case, since the same presentation happened in our patient, who survived previously treated malignancy. Herein, we present a case of single skull metastasis as the first presentation of metachronous lung adenocarcinoma, in a 54-year-old carcinoma of the head of the pancreas survivor following Whipple procedure performed five years before this presentation. Therefore, we aim in this report to describe this unusual case and raise the awareness of the development of metachronous primary lung cancer.

## 2. Case Report

The patient, a 54-year-old female, is a known case of carcinoma of the head of the pancreas, which revealed being a periampullary pancreatic adenocarcinoma after histopathological examination. She underwent uneventful Whipple's procedure in September 2010 followed by adjuvant chemotherapy (carboplatin and methotrexate), with the last cycle being five years before her presentation. She has been on regular follow-up with oncology department without local or systemic recurrence.

In November 2015, she presented with a progressive painless swelling in the right parietal region of six-month duration. There was no history of trauma or previous head radiation. Before the presentation, patient overall health condition was stable with unremarkable history. Her social history was unremarkable and specifically negative for smoking.

Her physical exam and vital signs were within normal. Local examination revealed a hard mass of 5 × 6 cm in diameter with distinct edges, fixed to the skull.

The scalp over it was mobile and with no discoloration or discharges and no regional cervical lymphadenopathy. Neurological examination revealed a conscious, alert, and oriented patient without a focal neurological deficit.

Laboratory workup including CBC, renal, hematology, and hepatic profiles was within normal. Chest X-ray identified a small nodule in the left upper lobe.

**Figure 1:**
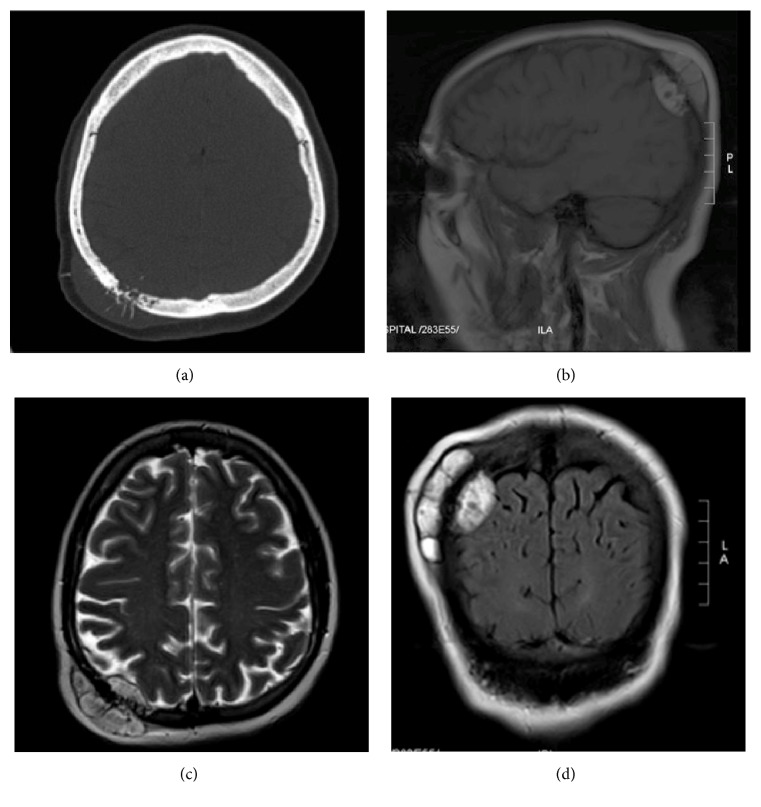
(a) Preoperative axial CT scan of the brain (bone window) showing osteolytic changes with associated soft tissue swelling. (b) Preoperative unenhanced parasagittal T1-weighted MRI image showing hyperintense epidural cystic mass compressing the cortex underlying. (c) Preoperative axial T2-weighted MRI image showing mildly hyperintense mass on the right parietal region. (d) Preoperative FLAIR coronal MRI showing hyperintense mass that traverses the skull.

Investigations also included CT brain, which demonstrated a lytic bony lesion with classic sunburst periosteal reaction measuring 4.3 × 3.1 × 5.4 cm (Figure 1(a)). The lesion was causing mild displacement of the adjunct cortex with no detected calcifications. There was no underlying vasogenic edema, and no other osseous skull lesions were detected. The cerebral and cerebellar parenchyma showed preserved gray-white matter differentiation, with no evidence of acute territorial infarction and no evidence of abnormally enhancing cerebral parenchyma or meninges. There was no midline shift, and ventricular system, basal cistern, and cortical sulci are within normal limits.

**Figure 2:**
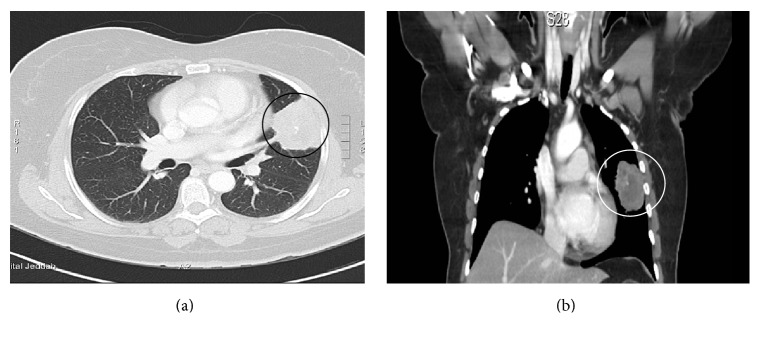
(a) Axial and (b) coronal contrast-enhanced chest CT scan showing malignant looking pleural-based lobulated left upper lobe soft tissue.

Magnetic resonance imaging (MRI) demonstrated a right parietal hyperintense lesion in nonenhanced T1- and T2-weighted image sequences which involved full skull thickness and has an epidural extension. The lesion demonstrates significant heterogenous enhancement after gadolinium administration, with adjunct focal dural enhancement. The gradient echo sequences demonstrate some areas of susceptibility artifacts within the lesion representing calcification (Figures 1(b), 1(c), and 1(d)). Further screening with CT scan of the chest and abdomen did not reveal any lesions aside from the lung nodule, which was malignant, looking like pleural-based lobulated left upper lobe soft tissue mass, with central necrosis measuring 4.1 × 5.3 cm (Figures 2(a) and 2(b)). Moreover, there were no additional lung lesions and no pleural effusion. Bone scan showed no evidence of other osseous metastases.

The provisional diagnosis was that the skull lesion was probably a metastatic deposit of the previously resected pancreatic cancer. Thus, surgery was planned to excise the skull lesion and send for histopathology, followed by CT guided biopsy of the lung nodule in a later date.

**Figure 3:**
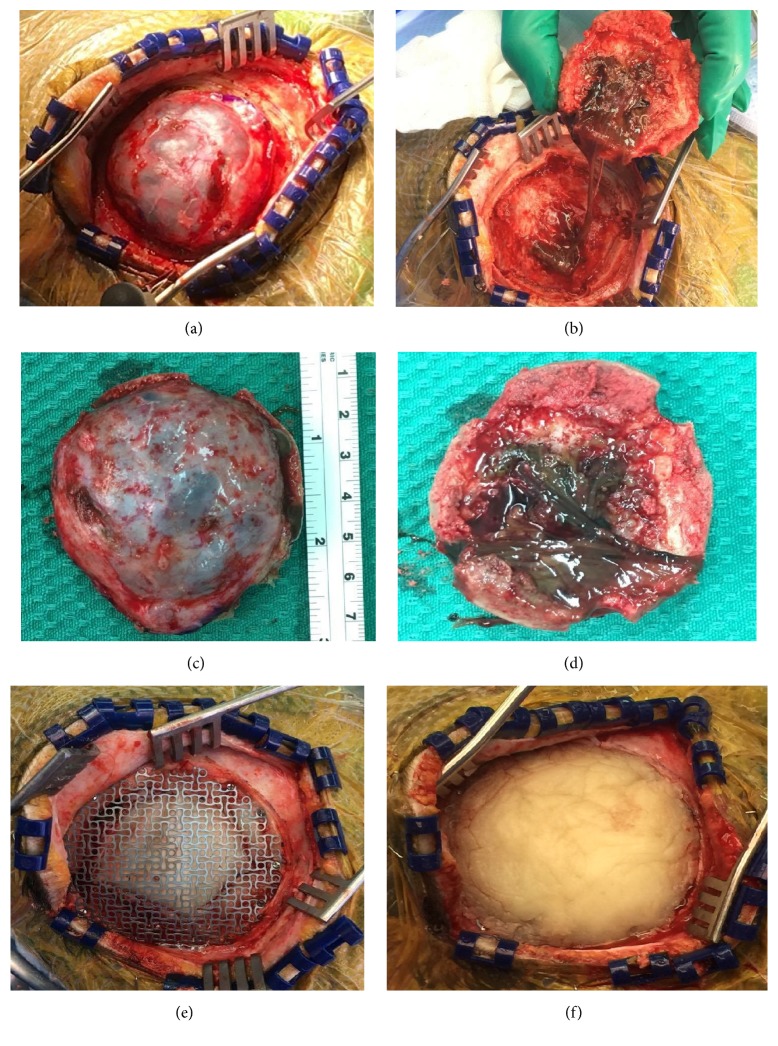
Intraoperative photographs showing (a) the cystic destructive skull lesion, (b) inner side of the lesion after reflection of bone flap, (c) outer side of lesion after excision, and (d) inner side of the lesion after excision. Duraplasty and the application of titanium mesh (e) followed by the application of cement (f).

Under general anesthesia, the patient was placed in a supine position, with the head fixed using three-pin Mayfield clamp. The patient was registered using neuronavigation, and anatomical marking of the tumor was achieved. After usual draping under aseptic condition, an incision was made in a straight fashion. The skull flap was reflected, and the lesion was identified without scalp involvement (Figure 3(a)). Two burr holes were created, and free craniotomy flap was raised with the precise demarcation of 2 cm from the tumor edges using the navigation. The entire lesion was excised; it was brown in color, slippery, and with high viscosity (Figures 3(b), 3(c), and 3(d)). The dura was excised up to the healthy looking edges of the craniotomy; after that, a dural graft measuring 4 × 4 cm was used to fill the dural defect created. The underlying brain tissue was normal without infiltration from the lesion. Titanium mesh was fixed, and cement was applied to fill the bony defect resulting from the craniotomy (Figures 3(e) and 3(f)). The excised lesion was sent for histopathology. The patient tolerated the procedures and the postoperative period was unremarkable.

**Figure 4:**
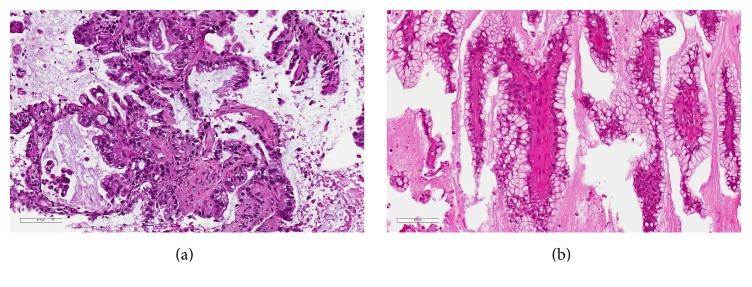
Both lung (a) and (b) skull bone and dura specimens demonstrating infiltrative glandular epithelium with intracytoplasmic mucin and hyperchromatic nuclei, some showing prominent nucleoli (hematoxylin & eosin, magnification ×200).

**Figure 5:**
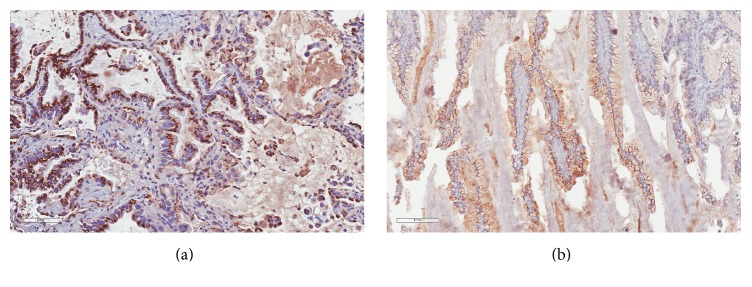
Tumor cells are positive for Napsin A staining in (a) lung and (b) skull bone and dura specimens (immunohistochemistry, magnification ×200).

Histopathological examination of the lesion revealed a piece of bone (5.5 cm × 5 cm × 1 cm) almost entirely replaced by a bulging mass that measures 1.5 cm in thickness. The cut surface is bone with cystic contents filled with mucinous material. Sections from bone revealed that a neoplastic growth was destructing and infiltrating in between bone trabeculae. The tumor infiltrated in form of glands lined by columnar epithelial cells with intracytoplasmic mucin. The nuclei are hyperchromatic and mildly pleomorphic with some showing prominent nucleoli (Figure 4). Immunohistochemistry is positive for Napsin A with basal cytoplasmic granular positivity pattern. It is also positive for cytokeratins CK7 and CK19 but negative for CK20, caudal type homeobox 2 (CDX2), and thyroid transcription factor-1 (TTF1) (Figure 5).

An issue was raised after histopathological examination that the skull lesion is mucinous in type, unlike the resected pancreatic adenocarcinoma, which was nonmucinous, raising the possibility that the solitary lung nodule is the source of this lesion as a second primary cancer.

The patient has been admitted again for CT guided biopsy of the lung nodule. Surprisingly, the obtained biopsy, after histopathological examination, showed infiltrative glandular epithelium with intracytoplasmic mucin, hyperchromatic nuclei, and prominent nucleoli, which is identical to the histopathological morphology of skull lesion, thus proving lung nodule to be a metachronous adenocarcinoma presenting initially with a skull metastasis. The sample of lung biopsy was tested for Epidermal Growth Factor Receptor (EGFR) status, and it was found to be of the wild type.

**Figure 6:**
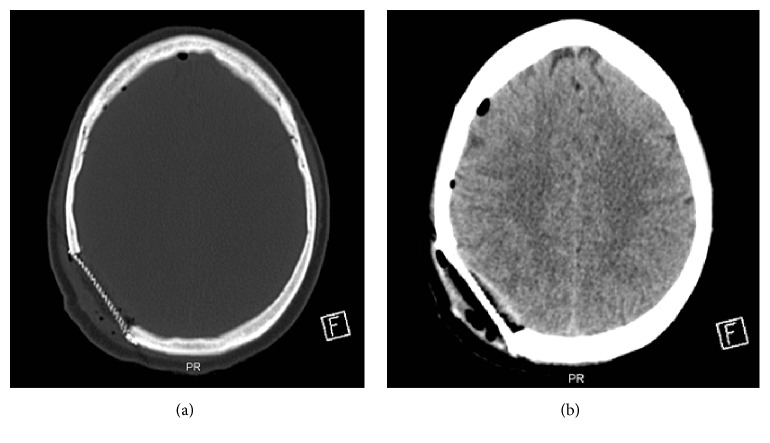
Postoperative nonenhanced (a) bone window and (b) brain window axial CT showing a metallic mesh over the site of bony resection, with no associated intracranial hemorrhage or residual bony lesion.

The patient had unremarkable recovery and received whole brain radiation 2 weeks later. The follow-up CT scan (Figure 6) revealed good reconstruction, and MRI scan revealed no recurrence at one-year follow-up. In regard to the treatment of the lung lesion, the patient received six cycles of chemotherapy (carboplatin and pemetrexed) for the primary lung adenocarcinoma, which failed to provide a tumor response. Then, she received three cycles of Docetaxel, which also failed too. Due to the poor response to chemotherapy, she underwent palliative radiotherapy for the lung mass, and she survives as of her last follow-up in June 2017 with stable lung disease.

## 3. Discussion

Certain types of cancers such as breast, prostate, and lung cancers are associated with high rate of metastatic deposits to other sites in the body [1]. A retrospective cohort study of 175 patients has found that 55% of skull metastases originated from breast origin, 14% from lung carcinoma, 6% from prostate cancer, and 25% from other origins [14].

Patients presenting with lung carcinoma are usually in an advanced primary disease stage, and survival of such population is poor and can be as low as 10–20% as reported by Stanley and his colleagues [1, 15]. Moreover, lung carcinoma usually metastasizes to bone rather than other sites, and rate of bone metastases can reach up to 36% comparing to metastases in other locations [2]. However, a small number of lung carcinomas metastasize to the skull, and they constitute, according to Turner et al., around 3% of total lung carcinoma bone metastases [2]. There is only one case reporting skull mass as the first presentation of lung adenocarcinoma in the literature [2], and, to our best knowledge, our reported case is considered the second. The uniqueness of our case from the previously mentioned similar reported case arises in that the skull swelling gave a hint about the development of rare entity such metachronous lung adenocarcinoma in a pancreatic cancer survivor, which had not been reported in the literature before.

Another unique feature found in the currently reported case is that the skull metastasis was originated from a metachronous lung adenocarcinoma that appeared after five years of carcinoma of the head of the pancreas resection. Such entity is rare in literature, and Schwarz et al. have reported three cases of secondary metachronous lung adenocarcinoma; in those cases, the time interval between the primary pancreatic carcinoma and secondary metachronous lung adenocarcinoma ranged from 16 to 66 months [16]. In our case, the patient developed secondary metachronous lung adenocarcinoma after 61 months of carcinoma of the head of the pancreas resection, which is considered a late metachronous adenocarcinoma, which falls into the time frame proposed by Schwarz et al.

Studies concerning genetic mutations that occur in such cases are lacking in the literature. However, there have been extensive reports discussing metachronous multiple primary tumors in the lung [17, 18]. Isaka et al. have demonstrated in their case report that epidermal growth factor receptor (EGFR) mutation status is an important indicator of determining the metachronous lung carcinoma, assisting in differentiating primary disease versus metastasis [17]. In a study including 59 sections of metachronous and synchronous lung carcinoma, it was found that 66% of the sections demonstrated mutations in* p53* gene, raising the possibility of an association between such mutations and the development of metachronous carcinoma [18]. Talbott and his colleagues, in their case series studying synchronous and metachronous cancer of the pancreas, have argued that K-ras mutations can play a role in the development of such circumstances [19]. Evidence in literature is still inconsistent, and other factors should be taken into account in determining genetic mutation abnormalities such types of cancers, although, with the limited occurrence of metachronous cancerous lesions, larger studies are required to provide a firm basis for profiling the genetic abnormalities of such cases.

Our case did not demonstrate the mode of spread of the skull metastasis from the lung adenocarcinoma. Even though metastases of lung carcinoma are widespreadly known to occur through the hematogenous spread, sometimes they appear in locations that are not predictable as a direct spread from the primary focus. Batson has proposed that vertebral veins could explain the appearance of cranial metastases from a lung origin, according to animal and cadaveric experimentation [20].

Skull metastases are usually small and asymptomatic and are discovered incidentally, thus posing a diagnostic difficulty. However, sometimes they are associated with pain, hemorrhage, or intracranial extension leading to focal neurological deficits [13, 21]. In some instances, dural sinuses become involved leading to rising in the ICP [13, 21].

Metastatic deposits to the skull are initially diagnosed by imaging such as CT and MRI [13, 22]. Usually, metastases from the lungs to the skull are lytic in nature causing bone destruction, same as lesions from breast, kidneys, and thyroid gland [13, 22]. In MRI, these lesions appear to be hypointense on T1-weighted images and with variable signal intensities in T2 images and variable enhancement by contrast injection [13, 22]. A study by Nemeth et al. has demonstrated that diffusion-weighted magnetic resonance imaging (DWI-MRI) is superior to routine MRI in detecting skull metastases from lung or breast origin [23]. Yohena and his colleagues argue that brain MRI is not to be done routinely in all lung carcinoma patients [24].

The definitive diagnosis is only available via histopathological examination coupled with immunohistochemistry (tissue diagnosis), as illustrated in our case since it can reveal an underlying, undiagnosed malignant disease [13]. The pathological specimen is usually taken after surgical excision, and sometimes due to the small size of the lesion or the presence of multiple lesions, fine-needle aspiration (FNA) is carried out [13, 25].

A significant number of patients with skull metastasis are not suitable for surgical intervention, owing in large part to the behavior of the primary malignancy and number of lesions [13, 26]. The difficulty of surgery depends on the involvement of vital structures such as the dural sinuses [13, 21]. Nevertheless, the surgical technique is straightforward involving the complete removal of the lesion and cranioplasty [13, 21]. In some instances, total excision of the lesion is not possible. Thus, radiotherapy is the another option for treatment [13, 26].

In a case of lung carcinoma, it has been suggested that lung carcinoma with distant metastasis is identified as a poor predictor of survival [1]. However, there have been many reports in which patients with solitary metastasis had an excellent increased rate of survival, with excision of the metastatic lesion and aggressive chemotherapy and radiotherapy [27, 28].

## 4. Conclusion

Skull metastasis from lung cancer is not uncommon, yet the first presentation for this malignant disease is a rare instant. The definitive diagnosis of the origin of metastasis is through tissue diagnosis. However, imaging has to be carried out to detect the primary malignancy and other metastases to guide surgical resection and subsequent treatment.
